# SANS-CNN: An automated machine learning technique for spaceflight associated neuro-ocular syndrome with astronaut imaging data

**DOI:** 10.1038/s41526-024-00364-w

**Published:** 2024-03-28

**Authors:** Sharif Amit Kamran, Khondker Fariha Hossain, Joshua Ong, Nasif Zaman, Ethan Waisberg, Phani Paladugu, Andrew G. Lee, Alireza Tavakkoli

**Affiliations:** 1 Human-Machine Perception Laboratory, Department of Computer Science and Engineering, University of Nevada, Reno, Reno, NV US; 2 Department of Ophthalmology and Visual Sciences, University of Michigan Kellogg Eye Center, Ann Arbor, MI US; 3 Department of Ophthalmology, University of Cambridge, Cambridge, UK; 4 Brigham and Women’s Hospital, Harvard Medical School, Boston, MA US; 5 Sidney Kimmel Medical College, Thomas Jefferson University, Philadelphia, PA US; 6 Center for Space Medicine, Baylor College of Medicine, Houston, TX US; 7 Department of Ophthalmology, Blanton Eye Institute, Houston Methodist Hospital, Houston, TX US; 8 The Houston Methodist Research Institute, Houston Methodist Hospital, Houston, TX US; 9 Departments of Ophthalmology, Neurology, and Neurosurgery, Weill Cornell Medicine, New York, NY US; 10 Department of Ophthalmology, University of Texas Medical Branch, Galveston, TX US; 11 University of Texas MD Anderson Cancer Center, Houston, TX US; 12 Texas A&M College of Medicine, Bryan, TX US; 13 Department of Ophthalmology, The University of Iowa Hospitals and Clinics, Iowa City, IA US

**Keywords:** Diseases, Medical research

## Abstract

Spaceflight associated neuro-ocular syndrome (SANS) is one of the largest physiologic barriers to spaceflight and requires evaluation and mitigation for future planetary missions. As the spaceflight environment is a clinically limited environment, the purpose of this research is to provide automated, early detection and prognosis of SANS with a machine learning model trained and validated on astronaut SANS optical coherence tomography (OCT) images. In this study, we present a lightweight convolutional neural network (CNN) incorporating an EfficientNet encoder for detecting SANS from OCT images titled “SANS-CNN.” We used 6303 OCT B-scan images for training/validation (80%/20% split) and 945 for testing with a combination of terrestrial images and astronaut SANS images for both testing and validation. SANS-CNN was validated with SANS images labeled by NASA to evaluate accuracy, specificity, and sensitivity. To evaluate real-world outcomes, two state-of-the-art pre-trained architectures were also employed on this dataset. We use GRAD-CAM to visualize activation maps of intermediate layers to test the interpretability of SANS-CNN’s prediction. SANS-CNN achieved 84.2% accuracy on the test set with an 85.6% specificity, 82.8% sensitivity, and 84.1% F1-score. Moreover, SANS-CNN outperforms two other state-of-the-art pre-trained architectures, ResNet50-v2 and MobileNet-v2, in accuracy by 21.4% and 13.1%, respectively. We also apply two class-activation map techniques to visualize critical SANS features perceived by the model. SANS-CNN represents a CNN model trained and validated with real astronaut OCT images, enabling fast and efficient prediction of SANS-like conditions for spaceflight missions beyond Earth’s orbit in which clinical and computational resources are extremely limited.

## Introduction

Spaceflight-associated neuro-ocular syndrome (SANS) refers to a unique constellation of neuro-ophthalmic imaging and clinical findings observed in astronauts after long duration spaceflight (LDSF)^1,2^. These findings include unilateral/asymmetric/bilateral optic disc edema of different Frisén grades, chorioretinal folds, posterior globe flattening, hyperopic refractive error shift, and cotton wool spots^3^. Although terrestrial analogs have been helpful in understanding SANS, there is no terrestrial equivalent and ongoing research is being conducted to further understand the underlying pathogenesis of this neuro-ophthalmic phenomenon^4–8^.

Assigned by the National Aeronautics and Space Administration (NASA), SANS has an elevated Likelihood and Consequence Ratio for a planetary mission to Mars, indicating potential long-term health consequences to astronauts, thus serving as one of the largest physiologic barriers to future spaceflight^9–11^. Mitigation research include lower body negative pressure^12,13^, pressurized goggles^14^, nutrition^15^, and understanding genetic predispositions to developing SANS^8^. While countermeasure efforts are promising, anticipated missions that expose astronauts to microgravity longer than what is known continue to require critical analysis of SANS progression. Moreover, anticipatory guidance for employing such countermeasures for SANS requires timely evaluation during deep-space exploration. On board the International Space Station (ISS), imaging modalities including optical coherence tomography (OCT), fundus imaging, and orbital ultrasound have been instrumental in providing high-quality analysis of SANS progression^3,16–18^. On deep space missions beyond Earth’s orbit, such as the mission to Mars and beyond, critically analyzing these images will be particularly challenging. Additionally, increases in transmission latency for these large data images to Earth may be a large barrier for an expert reader on Earth to critically analyze SANS progression in a timely fashion, particularly as spacecraft continues to travel farther from Earth^19–21^. This isolation from an expert evaluator of SANS progression during these missions may be mitigated with an automated, expert system that can analyze imaging data onboard the spacecraft. A primary consideration of such a system in the spaceflight environment is the limited computational capacity.

In this paper, we report the development, validation, and accuracy of a deep learning architecture with lightweight Convolutional Neural Networks (CNNs) designed specifically to detect SANS findings on OCT B-scans for future deep space exploration entitled “SANS-CNN”. We utilize SANS images provided and labeled by NASA to validate this architecture. We compare SANS-CNN to state-of-the-art, publicly available pre-trained architectures to interpret the robustness of such a system for spaceflight. The two primary aims of this architecture are: (1) to provide an accurate model that properly detects SANS-like features and (2) to utilize efficient and lightweight CNN predictions for the computationally limited spaceflight environment. The development of technology for detecting and evaluating SANS progression may serve to provide astronauts immediate guidance on whether escalation of SANS countermeasures (e.g., lower body pressure) is warranted during deep-space missions.

## Methodology

### Pre-trained Encoder

**Fig. 1:**
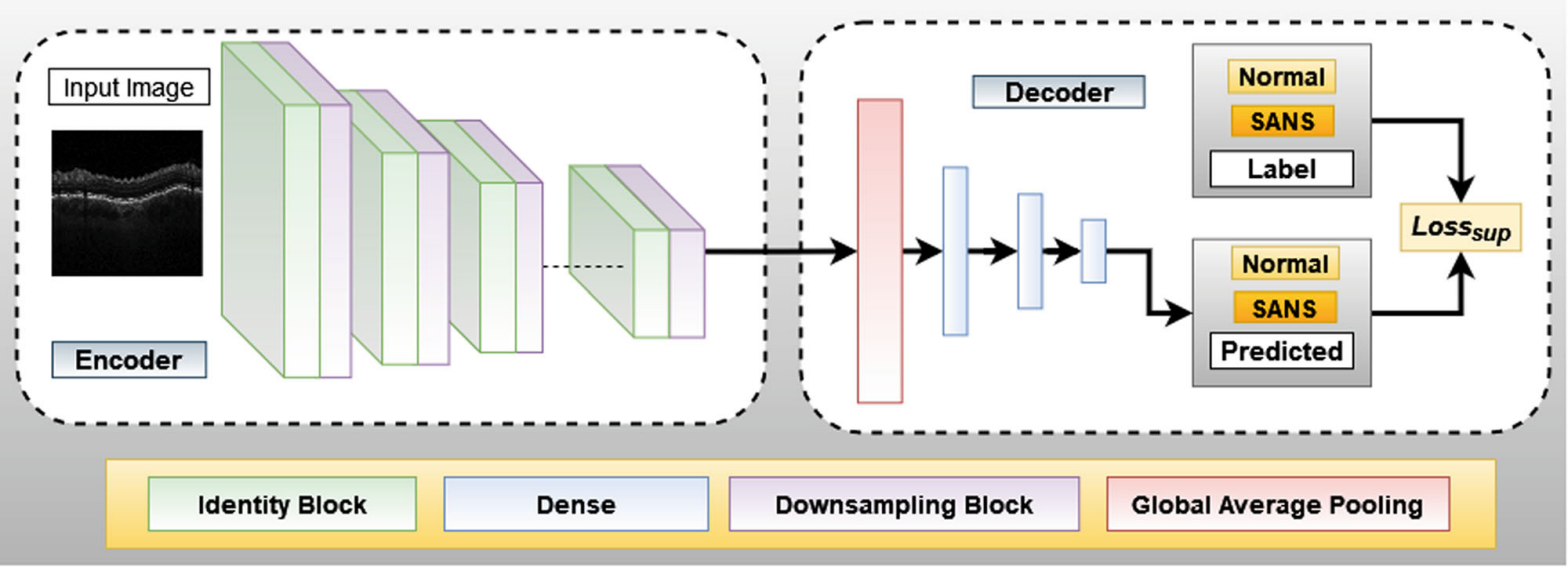
Proposed Deep Learning Architecture for SANS Classification. The encoder consists of Identity blocks which utilize convolution, batch-normalization and activation layers to learn inherent features and Downsampling blocks which down samples the spatial features to half the size using stride=2 convolution. The decoder consists of a Global average pooling layer to averaging the 2D feature in the depth axis and three Dense layers for flattening the 2D spatial features to 1D features. The labels utilized are “Normal” and “SANS”, and we utilize supervised cross-entropy loss function to train the model.

**Fig. 2:**
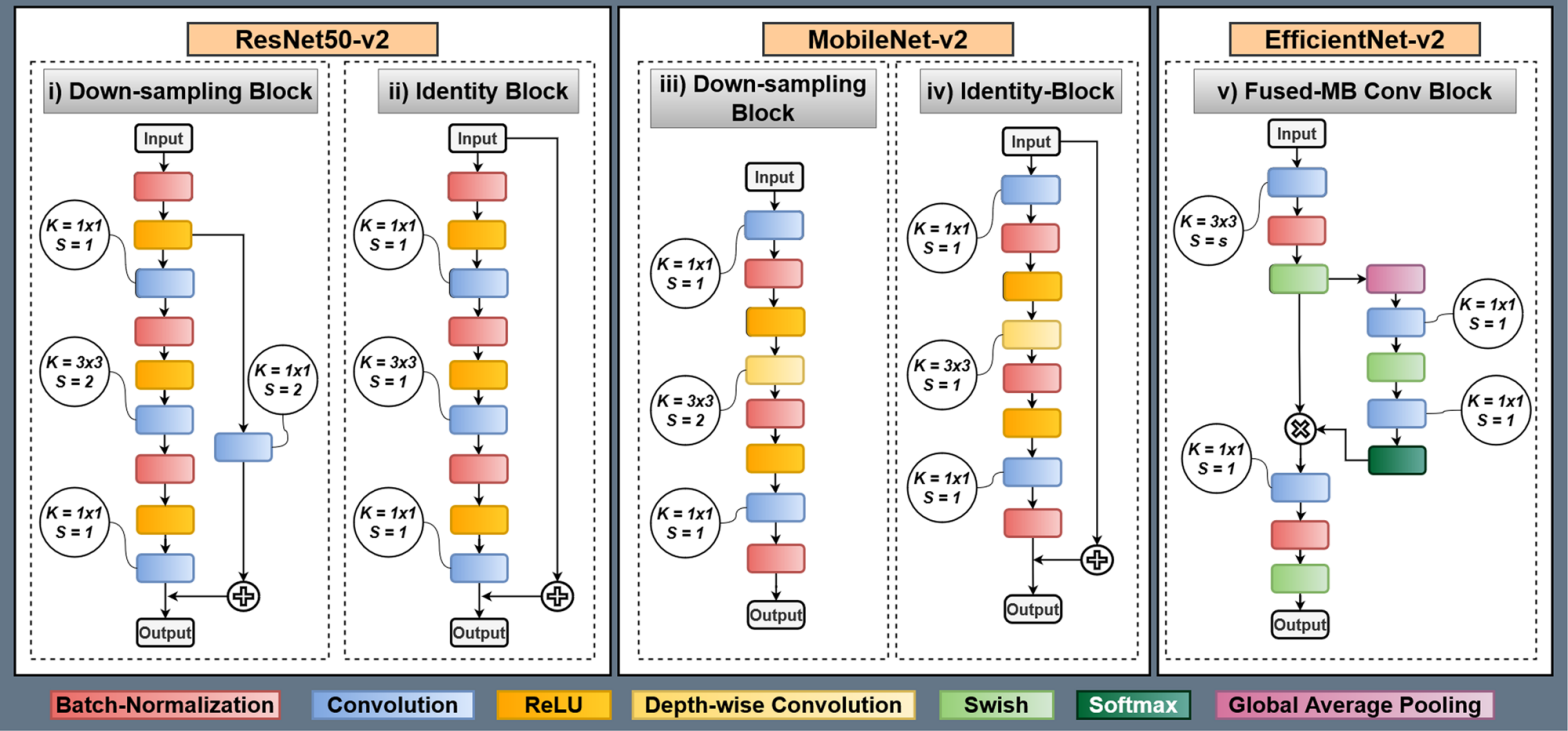
Building blocks of SANS-CNN which incorporates ResNet50-v2, MobileNet-v2 and EfficientNet-v2 identity blocks. ResNet50-v2 and MobileNet-v2 utilize separate learnable blocks for identity and down-sampling, whereas EfficientNet-v2 utilizes the same block for both. Here, K = kernel size, S = stride. ResNet50-v2 and EfficientNet-v2 have a skip connection in down-sampling block, but MobileNetV2 does not have a skip connection.

Utilizing pre-trained models for transfer learning tasks has shown tremendous promise in healthcare^22–24^, physics-driven simulation^25^, drug-discovery^26^, and computational biology^27^. Pre-trained models are architectures previously trained on a large data set, and then the weights of these models are transferred and trained on a downstream task. For example, a popular deep convolutional neural network called ResNet^28^ has been trained using 3.2 million ImageNet^29^ and images have been used for many downstream tasks for ophthalmology. Similar architectures with residual blocks have seen many adoptions in challenging downstream tasks for ophthalmology such as image-to-image translation, image inpainting, and image segmentation^30,31^. In Fig. 1, our proposed Efficient and Interpretable Deep ConvNet architecture is given, which consists of an encoder which takes the OCT B-Scans as input, a decoder, and an output for predicting between SANS and Normal cases. The encoder consists of a pre-trained network with multiple residual and downsampling blocks, as shown in Fig. 2.

The basic structure of ResNet consists of a residual unit with two consecutive convolution layers and a skip connection that adds the feature tensor of the input with the output^28^. However, the authors improved upon this, utilized pre-activation with batch-normalization to address the vanishing gradient problem, and proposed a new architecture called ResNet-v2 with new residual and downsampling blocks. In terms of efficiency, two other architectures have proposed modified learnable blocks which utilize fewer parameters: MobileNet^32^ and EfficientNet^33^. Similar to ResNet, the authors of both these models improved upon their architecture and proposed two new architectures, MobileNetV2^32^ and EfficientNetV2^33^. For our experimentation, we use pre-trained encoders of these three architectures. Moreover, all of them were trained on ImageNet2012 datasets^29^. The difference and similarities of these three architectures are given in Table 1.

**Table 1:** Comparison of ResNet50-v2, MobileNet-v2 and Efficient-v2 architectures regarding activation function, activation function placement, utilities distinct downsampling block, skip-connection operation, skip-connection in downsampling block, and presence of depth-wise convolution

	ResNet50-v2	MobileNet-v2	EfficientNet-v2
Activation function	ReLU	ReLU	Swish and Softmax
Activation function placement	Pre-activation	Post-activation	Post-activation
Utilities distinct downsampling block	Yes	Yes	No
Skip-connection operation	Element-wise Addition	Element-wise Addition	Element-wise multiplication
Skip-connection in downsampling block	Yes	No	Yes
Has depth-wise convolution	No	Yes	No

### Building Blocks

**Fig. 3:**
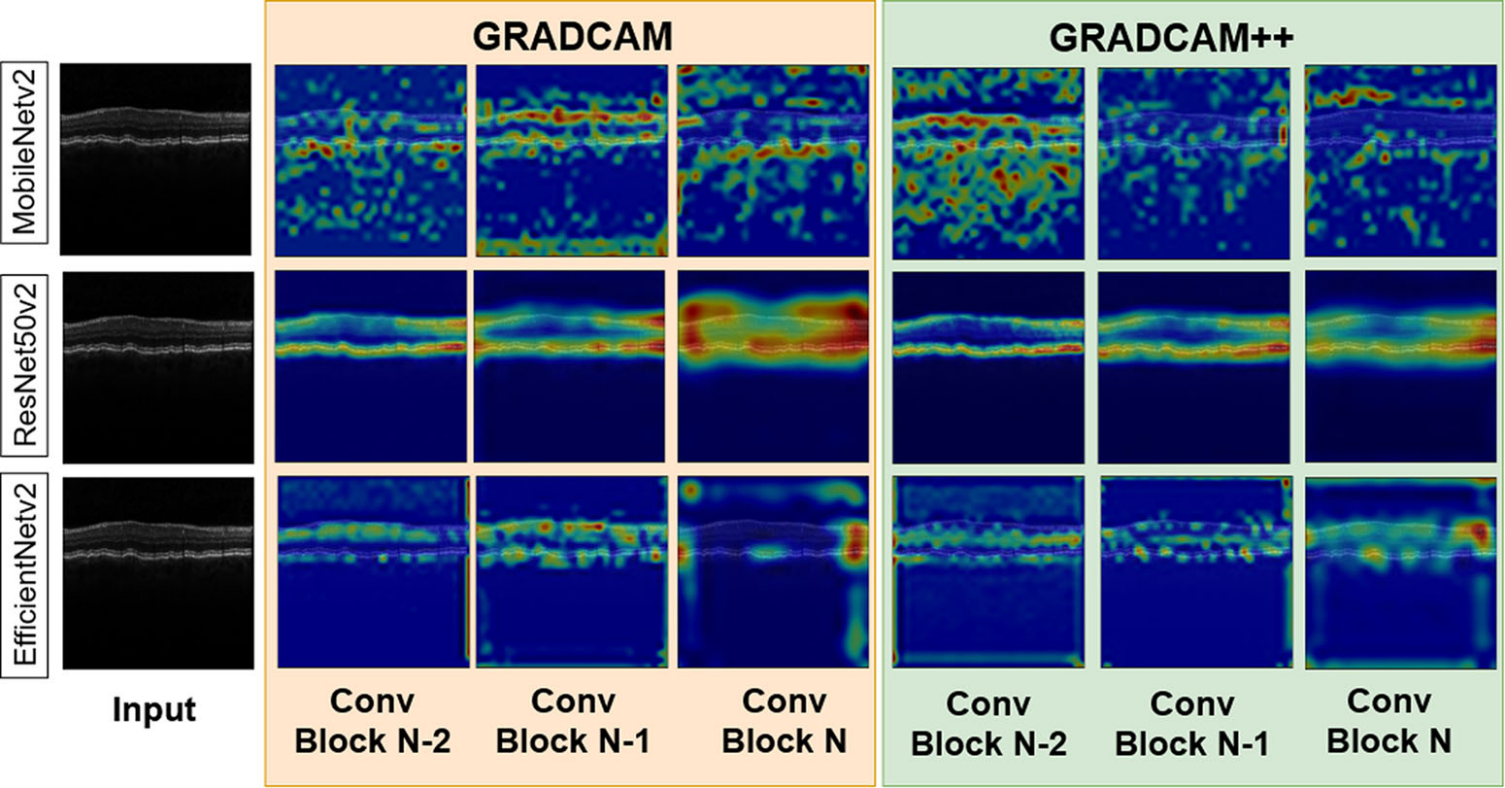
Visualization of back-propagated gradient activation maps using GRAD-CAM and GRAD-CAM++ on our three architectures. Here, Conv Block N is the output of the last block of the encoder, the Conv Block N-1 and Conv Block N-2 are preceding block’s outputs.

First, we use the ResNet50-v2 architecture for our pre-trained encoder. The encoder consists of residual downsampling and residual identity blocks with pre-activation. We illustrate both of these residual blocks in Fig. 3 (I) and (ii). The residual downsampling blocks have three sub-blocks successively with Batch-Normalization, ReLU, and Convolution. Also, a skip connection with the convolution layer is added from the first sub-blocks ReLU with the last convolution layer’s output. The first and last convolution has kernel size, *k* = *3* and stride, *s* = *1*. The second and skip-connection convolutions have stride, *s* = *2*. Kernel size and stride are utilized to determine the receptive field of the convolution operation to extract intrinsic spatial information from the images. We use a small kernel size and stride size to extract fine local features, which helps with better downstream information aggregation. On the other hand, downsampling layers are employed to decrease the image features spatially (typically half of the original by using stride = 2) for the convolution operation to work on, which helps with utilizing less memory. By combining small kernel and stride size and using downsampling after each stage, we minimize the overall number of parameters, which effectively helps with overall memory utilization and redundant feature extraction.

Next, we use the MobileNetV2 pre-trained encoder, consisting of downsampling and identity blocks. Both of these blocks are visualized in Fig. 3 (iii) and (iv). Unlike ResNetV2, the MobileNetV2 uses post-activation. Both downsampling and identity blocks consist of three sub-blocks. The first and last sub-blocks have Convolution, Batch-normalization, and ReLU activation layers. The second sub-block has Depth-wise Convolution, Batch-normalization, and ReLU activation layers. The identity block has a skip connection from the input and is added with the output of the last ReLU layer. The first and last convolution has kernel size, *k* = *3* and stride, *s* = *1*. The depth-wise convolution has stride, *s* = *2*.

Lastly, we incorporate the EfficientNetV2 pre-trained encoder, which consists of Squeeze-and-excitation blocks. The block is illustrated in Fig. 3(v). The block consists of three sub-blocks, with the first and third consisting of Convolution, Batch-Normalization, and Swish activation layers. The second sub-block consists of Global-average pooling, Convolution, Swish activation, Convolution, and Softmax activation layers. The output of the second sub-block is element-wise multiplied by the first sub-block’s output. The convolution in the sub-block has a kernel size, *k* = *3*, and the rest of the convolution in other sub-blocks has a kernel size, *k* = *1*. As Efficient-NetV2 only utilizes this block for downsampling and regular blocks, the stride size is changed in the first sub-block depending on the block definition. The stride size is chosen as, *s* = *2* for downsampling and *s* = *1* for a regular block.

### Decoder

The decoder consists of a global average pooling layer, followed by three dense layers as illustrated in Fig. 2. The global average pooling takes the average of the spatial dimensions and transforms it into a 1D feature vector. The three dense layers consist of 256, 64, and 2 neurons. The first two dense layers are followed by a dropout layer which randomly drops activations to zero. We use drop rate of 0.6 and 0.2 (out of 1) for the dropout layers successively. We use two neurons in the last layer for predicting SANS or Normal class. The downstream task is a supervised-classification task, as the ground-truth values of one hot-encoded vector.

### Objective function

For training we incorporate weighted categorical cross-entropy loss as shown in Equation (1). Here, ${y}_{i}$ is the ground-truth class and ${y}_{i}^{{\prime} }$ is the predicted class. The $c$ signifies the number of samples, ${w}_{i}$ signifies weight values, and ${cat}$ denotes that is a categorical loss. The weight values were chosen based on the number of samples per-class.

$${{\mathscr{L}}}_{{w}_{{cat}}}=-\mathop{\sum }\limits_{i=1}^{c}{w}_{i}{y}_{i}\log \left({y}_{i}^{{\prime} }\right)$$

**Equation 1**. Weighted categorical cross-entropy loss.

### Data preprocessing

For our experimentation, we use the OCT-B scans that are separated for training, validation and testing based on unique astronauts. The control images are from pre-flight, in-flight and post-flight OCT volumes. The SANS images were taken from both in-flight and post-flight OCT volumes. For training, we utilized 2506 SANS and 3797 Normal OCT B-scans. For validation, we incorporated 627 SANS and 950 Normal OCT B-scans. In order to evaluate on a hold-out test set, we utilized 467 SANS and 478 Normal images. All images were normalized to have values between 0–1 from 0–255-pixel intensities for training, validation and testing and the images were pre-processed to have a resolution size of 512 × 512.

### Hyper-parameter Tuning

For training all the models, we used Adam optimizer for adapting the parameter learning rates^34^. The initial learning rate, we used the Adam optimizer with an initial learning rate of ${\rm{lr}}=0.0001.$ We utilized a mini-batch of $b=16$ and trained all methods for 30 epochs. For the class weights, we chose 0.83 for majority (Normal) and 1.257 for minority (SANS) training samples. We used the Keras Deep learning library (https://keras.io/) with Tensorflow backend (https://www.tensorflow.org/) to train our models. For training, we utilized two callbacks: “Reduce Learning rate on Plateau” and “Model Checkpointer”. The first callback reduced the learning rate by $0.1$ if the validation loss did not decrease for six epochs. Whereas the other callback saves the best snapshot of the model weights for each epoch.

### Metrics

We used three standard metrics for calculating the Accuracy, Sensitivity (True Positive Rate), Specificity (True Negative Rate), Precision (Positive Predictive Value) and F1-score. The metrics are calculated as follows in Equation (2):

$${\rm{Accuracy}}=\frac{1}{N}\sum \frac{{\rm{TP}}+{\rm{TN}}}{{\rm{TP}}+{\rm{TN}}+{\rm{FN}}+{\rm{FP}}}$$

$${\rm{Sensitivity}}=\frac{1}{K}\sum \frac{{TP}}{{TP}+{FN}}$$

$${\rm{Specificity}}=\frac{1}{K}\sum \frac{{TN}}{{TN}+{FP}}$$

$${\rm{Precision}}=\frac{1}{K}\sum \frac{{TP}}{{TP}+{FP}}$$

$${\rm{F}}1-{\rm{score}}=\frac{1}{K}\sum \frac{2{TP}}{2{TP}+{FP}+{FN}}.$$

**Equation 2**. Equations to calculate accuracy, sensitivity, specificity, precision, and F1-score. Here, N is the number of samples, and K is the number of classes (K = 2). TP= True Positive, FP = False Positive, FN= False Negative, TN = True Negative.

## Results

### Quantitative results

As seen in Table 2, the best-performing model, EfficientNet-v2, achieved 84.2% accuracy, 85.6% specificity, 82.8% sensitivity, and an 84.1% F1-score. Compared to that, ResNet50-v2 achieved differences of 17–23% less across all six metrics. In contrast, MobileNet-v2 achieved a difference of 12.1% improvement in sensitivity but got lower precision, specificity, F1-score and accuracy. It is evident that EfficientNet-v2 has better overall performance in this SANS vs. Normal images recognition task.

**Table 2:** Performance comparison of deep learning models on SANS and Normal OCT B-scans

Model	Accuracy	Sensitivity	Specificity	Precision	F1-score
EfficientNet-v2	84.2%	82.8%	85.6%	85.5%	84.1%
MobileNet-v2	71.1%	94.9%	46.6%	64.5%	76.8%
ResNet50-v2	62.8%	63.1%	62.5%	63.3%	63.2%

### Qualitative results

For producing “visual justifications” for decisions made by our ConvNet models for accurate classification, we use GRAD-CAM^35^, and GRAD-CAM++^36^. These techniques utilize back-propagated gradients to show important regions of the images from the perspective of a specific layer, intensified for the classification decision’s maximum probability. In Fig. 3, we visualize the differences in activations of three blocks of the encoder layers for three of our models on a SANS image. Stage N is the last convolution blocks’ output before the global average pooling layer. Similarly, Stage N-1 and Stage N-2 are the previous convolution block’s outputs. From Fig. 1, row 1, it is apparent that MobileNetV2 had sparsely activated signals in choroidal fold region. However, in row 2, ResNet50v2 had concentrated activated signals and activations in the choroidal folds and the unimportant region of Bruch’s Membrane (BM) and retinal pigment epithelium (RPE). In contrast, our best architecture, EfficientNetV2, had the best output for choroidal folds manifested in the RPE and BM as well as the cotton wool spots manifesting below the retinal nerve fiber layer (RNFL). This visualization conforms to the patterns seen in the output metrics where EfficientNevV2 performs the best in most metrics while ResNet50v2 is the lowest performing. This qualitative illustration compliments our model’s overall explainability and knowledge transferability.

## Discussion

The results from SANS-CNN demonstrate a robust detection of SANS findings with lightweight CNNs compared to state-of-the-art pre-trained architectures. Given that prolonged optic disc edema and chorioretinal folds have been observed to lead to potential significant visual impairment terrestrially (e.g., chorioretinal folds-related maculopathy or idiopathic intracranial hypertension)^37–40^, the automatic evaluation of SANS for immediate insight to onboard members is critical for deep space exploration. As mitigation strategies continue to develop for SANS, there is a necessity for guidance on how long astronauts should employ these countermeasures during spaceflight. Countermeasures such as LBNP and goggles have shown promise for counteracting SANS^13,14^. LBNP attenuates the cephalad fluid shifts during spaceflight that result from a reduction in hydrostatic pressure in microgravity. These cephalad fluid shifts have been hypothesized to be a critical component to SANS development. In a randomized crossover trial in a terrestrial setting, Hearon et al. observed that LBNP at night mitigated an increase in choroidal area and volume measured by OCT during supine bed rest, suggesting that this countermeasure during sleep on spaceflight missions may counteract SANS. During prolonged exposure to microgravity than what is known, SANS may continue to progress despite nightly LBNP. Automated and timely evaluation of SANS may provide guidance to whether further escalation of lower body negative pressure beyond just nightly use is necessary to attenuate SANS progression.

The utilization of SANS-CNN can be further validated with terrestrial analogs of SANS. Head-down tilt bed rest is a promising terrestrial analog of SANS which mimics cephalad fluid shifts in spaceflight and has been observed to produce optic disc edema and chorioretinal folds^41^. In conjunction with countermeasures during head-down tilt bed rest, SANS-CNN may help to provide an objective evaluation of SANS-like progression with terrestrial analogs.

Several limitations exist for this study that may be considered for future technical development. First, the labelled OCT data provided by NASA spans across many years with different evaluating experts which limits the uniformity of data. This can be addressed with prospective studies in SANS with uniform labelers. Secondly, given the natural limitation of humans that go to space and the relatively recent discovery of SANS, the number of SANS images may be increased in the future to provide a more robust training dataset. This limitation can be approached with deep learning transfer learning techniques that can build upon larger datasets with terrestrial data to strengthen a smaller training dataset^42,43^. However, SANS has no terrestrial equivalent; thus, utilizing astronaut SANS images from future spaceflights will be of utmost importance. Though we achieved over 80–85% on five different metrics, there is room for improvement to enhance the architecture to achieve accuracies above 95%. Given, the model is receiving OCT B-scans image inputs for healthy and SANS patients, some B-scans from the SANS patients might not contain apparent SANS indicator (like choroidal folds or cotton wool spots), which might explain the model’s score not reaching 90% or beyond. In the future, our team seeks to investigate whether pre-training on non-SANS publicly available retinal degenerative images (e.g., age-related macular degeneration and diabetic macular edema) can improve the overall performance of our model. Additionally, we seek to explore the incorporation of Generative Adversarial Networks to generate synthetic SANS data to improve the model further^20,44,45^. Lastly, in the extraterrestrial environment with limited resources such as an exploratory mission traveling away from Earth, optimizing computational utilization and lightweight aspects of such an automated system is critical for systems resourcefulness. Thus, continuing to optimize the computational efficiency of this system is of utmost importance.

Automated detection of SANS during deep-space exploration where clinical resources are limited may provide necessary and timely guidance on countermeasure utilization. SANS-CNN is designed specifically for astronaut ophthalmic health and demonstrates robust detection of SANS-like conditions when compared to existing state-of-the-art architectures. This work is a part of a comprehensive ocular framework to monitor SANS with deep learning and extended reality^46–51^. Future directions include developing and merging models with other modalities onboard the ISS such as fundus photography, which is useful for detecting optic disc edema, chorioretinal folds, and cotton wool spots. As OCTA information becomes available, labelled data with SANS can be merged within SANS-CNN to provide even more robust SANS detection with additional choroidal vasculature data. Further research of SANS-CNN includes employing additional terrestrial neuro-ocular pathologies (e.g., papilledema in idiopathic intracranial hypertension) to further understand and delineate the similarities, differences, and spatial features of this unique microgravity phenomenon from terrestrial diseases. This may also help to further understand the etiology of SANS and its possible multi-factorial pathogenesis. Further prospective research with this machine learning model, including comparing astronaut terrestrial data to their SANS data, may further our understanding and ability to detect SANS.

## Data Availability

Due to the nature of the research, the supporting astronaut data is not available.

## Code availability

Code available upon reasonable request.
